# Sociodemographic characteristics associated with adolescent depression in urban and rural areas of Hubei province: a cross-sectional analysis

**DOI:** 10.1186/s12888-019-2380-4

**Published:** 2019-12-05

**Authors:** Guo Li, Junhua Mei, Jing You, Jinfeng Miao, Xiaoyan Song, Wenzhe Sun, Yan Lan, Xiuli Qiu, Zhou Zhu

**Affiliations:** 1 Department of Neurology, Tongji Hospital, Tongji Medical College, Huazhong University of Science and Technology, 1095 Jiefang Avenue, Wuhan, 430030 Hubei China; 2 Department of Neurology, Wuhan First Hospital, 215 Zhongshan Avenue, Wuhan, 430030 Hubei China; 3 Blue Sky Women and Children Rights Protection Association, 96 Jingnan Avenue, Jianli, 433300 Hubei China

**Keywords:** Adolescent depression, Nomogram, Urban area, Rural area, Sociodemographic characteristic

## Abstract

**Background:**

China has experienced rapid socioeconomic, and health transitions over the last four decades, and urban–rural disparities are becoming increasingly apparent. Research on depression among rural and urban students can provide evidence on the relationship between sociodemographic characteristics and adolescent depression.

**Methods:**

We examined the association between sociodemographic characteristics and adolescent depression among 3605 students from Wuhan city and Jianli county that was recruited from the local junior middle school via a cross-sectional study. Univariate and multivariate logistic regression models were used to explore the sociodemographic characteristics of adolescent depression in urban and rural areas, respectively. Nomograms were constructed to calculate individual depression risk of junior middle school students.

**Results:**

32.47% of rural students and 35.11% of urban students display depressive symptoms. The protective factors of depression in urban students are exercise habit, younger, key class, better academic achievement and males, while Left-behind children (LBC), poor academic achievement and females had higher depression risk in rural area. Two nomograms were constructed to screen the adolescent depression in urban and rural junior middle school students, respectively. The clinical tools were well calibrated.

**Conclusion:**

The field-based research examined sociodemographic characteristics potentially associated with adolescent depression and offered an effective and convenient tool of individualized depression risk evaluation for junior middle school students. Future longitudinal epidemiologic research on adolescent depression may help to further validate the discovery of present study, which will support developing policies and practices to minimize the factors of adolescent depression.

## Background

Depression in adolescence is a public health concern worldwide and associated with substance abuse, academic problems, cigarette smoking, physical health problems, impaired social relationships and suicide [1]. Previous epidemiological and clinical researches have indicated that adolescent depression has numerous individual, family and social factors. Reported risk factors for adolescent depression include being female, anxious, offspring of depressed parents, and being exposed to stress or trauma [2–4]. However, many of the known contributing factors have been described in the western context. Since adolescents are psychologically vulnerable to the influence of their family and social surroundings, sociodemographic characteristics for adolescent depression may vary across countries, or even vary across regions [5]. China, in particular, has experienced rapid socioeconomic, and health transitions over the last four decades. These huge changes have made China a unique and important object for adolescent depression research.

Meanwhile, given there is large inequality in socio-economic, policy and cultural environment between rural and urban areas in China, research examining the relationship between sociodemographic characteristics and adolescent depression in a specific social context of China is needed. Although some studies have investigated sociodemographic characteristics related to depression in Chinese adolescents [6–9], few researches have been conducted to systematically investigate the psychological health contrast of adolescents from urban and rural areas [10–12]. Urban–rural mental health disparities are expected to be even more pronounced in China, since they have important social values and have been well studied in developed countries [13–15]. Research on depression among rural and urban students can provide evidence on the relationship between depression and sociodemographic status in this unique cultural setting, and may be helpful to develop policies and practices to minimize the risk factors of adolescent depression. In this study, we choose Wuhan city and Jianli county as the objects of urban-rural depression disparity research. Wuhan is the provincial capital of Hubei province and the largest city in central China. Wuhan ranked the 9th in Chinese cities in terms of Gross Domestic Product (GDP) in 2018 based on the National bureau of statistics. Jianli county, however, is one of the most economically undeveloped regions in Hubei province. Nearly 500,000 migrant workers are working outside the city, making left-behind children and depression in adolescence serious public health problems in local area. Therefore, the two regions targeted in this study are appropriate for urban-rural depression disparities research.

Furthermore, as a result of the traditional Chinese culture’s strong stigma toward psychiatric disorders, the serious shortage of psychiatrists and infrastructure (especially in the rural areas) and low mental health literacy, only 8.3 to 27.6% of patients with depressive disorders are diagnosed, and about half of those diagnosed receive effective treatment [16]. Adolescence is the stage in which most mental health problems first manifest [17]. Researches also suggest that delays in seeking help for mental health problems lead to poorer treatment outcomes [18, 19]. Knowledge of differences in risk factors of psychological health can aid in the development of targeted student’s mental health education. In addition, we also aim to develop effective, easy-to-use clinical screening tools to identify high-risk groups of adolescent depression.

A cross-sectional study was therefore designed to find the potential risk factors and their differences between urban and rural areas. We aim to examine the potential relationship between sociodemographic characteristics and depressive symptoms among rural and urban junior middle school students and construct nomograms to predict the risk of adolescent depression based on the selected factors.

## Methods

### Study sites and population

**Fig. 1:**
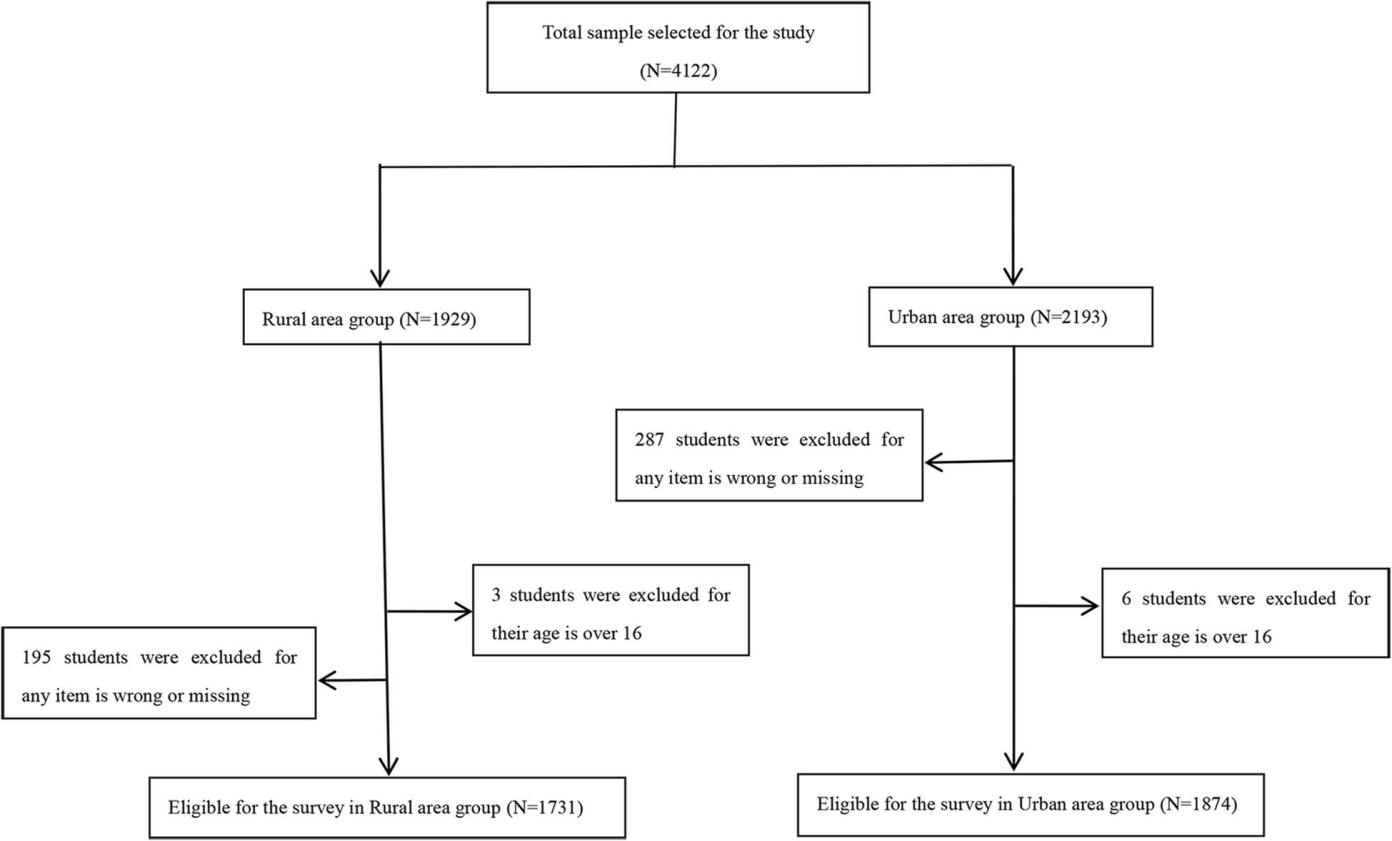
Flow chart of sample selection according to inclusion and exclusion criteria

The study was conducted between November 2018 and February 2019 in Wuhan city and Jianli county, Hubei Province. Three junior middle schools of Wuhan city and four junior middle schools of Jianli county were selected with stratified-cluster sampling method and 4122 junior high school students were included covering three grades. 3605 students aged from 11 to 15 years old completed the survey (Fig. 1).

### Data collection

The investigation was organized and coordinated by Department of Neurology, Tongji Hospital, Tongji Medical College, Huazhong University of Science and Technology. The investigators who conducted the questionnaire survey were trained uniformly. The senior investigators checked the collected questionnaires daily to perform quality control. Data were entered double-blindly into the database by two different researchers using Epidata 3.0 to guarantee accuracy.

### Measures

The outcome for this study was confirmation of depression which was judged by the Center for Epidemiology Studies Depression Scale (CES-D Scale). The Chinese version of CES-D Scale has been used in previous research and its reliability and validity has been tested among Chinese populations. The depressed students were determined based on CES-D scores ≥20. The Global Physical Activity Questionnaire was used for the assessment of exercise habits [20, 21]. The independent variables were mainly applied to describe the characteristics of the students and their families. Specifically, the data allow us to generate variables that measure personal characteristics, including gender, age, only child status, academic achievement, class status, exercise and boarding status; and household characteristics, including three-generational household, father’s and mother’s education level, left behind children (LBC) and single parent family.

LBC refer to the children have rural hukous and resided in rural areas while their parents both worked and lived outside of the household for at least 5 months out of the previous year. A single parent family refers to a family with children that were brought up by a single parent. Exercise habits are defined as meeting the WHO physical activity recommendations which children and youth aged 5–17 years old should accumulate at least 60 min of moderate to vigorous intensity physical activity daily, and most of the daily physical activity should be aerobic, at least 3 times per week.

### Statistical analyses

Continuous data were presented as the medians and ranges and compared using Mann Whitney U test. Categorical data were shown as frequencies and proportions and compared by Chi-square test or Fisher’s exact test. Risk factors to predict depression risk selected for the derivation of screening models were based on previous publications, which were routinely accessible in clinics with high measurement accuracy. Variables with a *P* value less than 0.05 in univariate analyses were subjected to multivariable Logistic regression analysis. Statistical analyses to identify risk factors were performed using SPSS software version 22.0 (Statistical Package for the Social Sciences) for Windows (SPSS, Chicago, IL). A nomogram was formulated based on the results of multivariate analysis, by using the R package “rms” in R software version 3.5.2 (http://www.r-project.org/). A final model selection was performed by a backward selection process based on the Akaike information criterion (AIC). The performance of the nomogram was measured by concordance index (C-index), equivalent to area under the curve value (AUC), and assessed by comparing nomogram-predicted probability versus observed probability. Bootstraps with 200 resamples were applied to these activities. The C-index reflected the probability that a randomly selected student with higher depression risk predicted via the nomogram more likely to be depressive than another randomly selected student with lower predicted probability. A higher C-index indicates better ability to separate junior school students with different depression risk. The calibration curves were used to compare the predicted probability with the observed probability in the study. If the model calibration is correct, dots on the calibration plot should be close to a 45° diagonal line.

## Results

### Sample characteristics at baseline

Sample characteristics for the total sample (*n* = 3605) and those rural students (*n* = 1731) vs. urban students (*n* = 1874) at baseline were presented in Table 1. The comparison of social characteristics of the depressed and non-depressed students were presented in Table 2. The Cronbach's α coefficients of the demographic questionnaire and CES-D Scale were 0.82 and 0.88.

**Table 1:** Characteristics of study subjects of urban or rural junior middle school

Variables	Total (*N* = 3605)	Rural (*N* = 1731)	Urban (*N* = 1874)	*P*
Age, years (Median, IQR)	13 (13~14)	13 (12~13)	14 (13~14)	0.00
Females, n (%)	1560 (43.27)	657 (37.95)	903 (48.19)	0.00
Depression, n (%)	1220 (33.84)	562 (32.47)	658 (35.11)	0.094
Accommodation, n (%)				0.00
At home	2393 (66.38)	580 (33.50)	1813 (96.74)	
In residence	735 (20.39)	709 (40.96)	26 (1.39)	
Others	477 (13.23)	442 (25.53)	35 (1.87)	
Academic achievement, n (%)				0.00
Excellent	567 (15.73)	171 (9.88)	396 (21.13)	
Average	1322 (36.67)	627 (36.22)	695 (37.09)	
Poor	1716 (47.60)	933 (53.90)	783 (41.78)	
Key class, n (%)	1547 (42.91)	476 (27.50)	1071 (57.15)	0.00
Three-generational household, n (%)	847 (23.50)	342 (19.76)	505 (26.95)	0.00
Only children, n (%)	2208 (61.25)	260 (15.02)	1137 (60.67)	0.00
Left behind children, n (%)	1041 (28.88)	887 (51.24)	154 (8.22)	0.00
Single parent family, n (%)	210 (5.83)	90 (5.20)	120 (6.4)	0.123
Full-time mother, n (%)	579 (16.06)	316 (18.26)	263 (14.03)	0.001
Father’s education level, n (%)				0.00
Primary school or below	479 (13.29)	352 (20.34)	127 (6.78)	
Secondary school	2075 (57.56)	1257 (72.62)	818 (43.65)	
College degree or above	1051 (29.15)	122 (7.04)	929 (49.57)	
Mother’s education level, n (%)				0.00
Primary school or below	810 (22.47)	667 (38.53)	143 (7.63)	
Secondary school	1863 (51.68)	979 (56.56)	884 (47.17)	
College degree or above	932 (25.85)	85 (4.91)	847 (45.20)	
Exercise habit, n (%)	1735 (48.13)	666 (38.47)	1069 (57.04)	0.00

**Table 2:** Characteristics of study subjects with or without depression

Variables	Depressed (*N* = 1220)	Non-depressed (*N* = 2385)	*P*
Age, years (median, IQR)	14 (13~14)	13 (13~14)	0.000
Females, n (%)	614 (50.33)	946 (39.66)	0.000
Urban areas, n (%)	658 (53.93)	1216 (50.99)	0.094
Accommodation, n, %			0.873
At home	816 (66.89)	1577 (66.12)	
In residence	247 (20.25)	488 (20.46)	
Others	157 (12.87)	320 (13.42)	
Academic achievement, n (%)			0.000
Excellent	152 (12.46)	415 (17.40)	
Average	343 (28.11)	949 (39.79)	
Poor	695 (56.97)	1022 (42.85)	
Key class, n (%)	563 (46.15)	984 (41.26)	0.005
Three-generational household, n (%)	929 (76.15)	1829 (76.69)	0.717
Only children, n (%)	494 (40.49)	903 (37.86)	0.125
Left behind children, n (%)	376 (30.82)	665 (27.88)	0.066
Single parent family, n (%)	88 (7.21)	122 (5.12)	0.011
Full-time mother, n (%)	208 (17.05)	371 (15.56)	0.248
Father’s education level, n (%)			0.028
Primary school or below	184 (15.08)	295 (12.37)	
Secondary school	670 (54.92)	1405 (58.91)	
College degree or above	366 (30.00)	685 (28.72)	
Mother’s education level, n (%)			0.630
Primary school or below	273 (22.38)	539 (22.60)	
Secondary school	620 (50.82)	1243 (52.12)	
College degree or above	327 (26.80)	605 (25.37)	
Exercise habit, n (%)	480 (39.34)	1255 (52.62)	0.000

52.00% of students live in urban areas and 48.02% of students lived in rural areas. Urban and rural students had significant differences in the following demographic variables: gender, only child status, class status, exercise, boarding status, three-generational household, father’s education level, mother’s education level, full-time mother, LBC and exercise habit. In rural samples, 51.24% of students are LBCs, 62.04% of the students are males and 40.96% of students boarded at school, which are significantly higher than urban counterparts. As well, 15.02% of rural students were the only children, 7.04% of the fathers and 4.91% mothers have a college education level or above, and 38.47% of rural students have exercise habits, which are significantly lower than urban counterparts. In summary, students from rural areas are younger (*P* = 0.00), with a higher proportion of males (*P* = 0.00), underachieving-students (*P* = 0.00) and residential students (*P* = 0.00), and are more likely to be raised by full-time mother than urban counterparts (*P* = 0.001). Students from urban areas were more likely from one-child families (*P* = 0.00) and three-generational household families (*P* = 0.00), studying in key classes (*P* = 0.00) and having exercise habits (*P* = 0.00) than rural counterparts. As well, their patents of the urban students are better educated (both *P* values = 0.00)

The comparison results of the depressed and non-depressed groups revealed that the adolescents in depressed group were older (*P* = 0.00), with more females (*P* = 0.00) and key class students (*P* = 0.005). The adolescents in depressed group were more likely from single parent family (*P* = 0.011), with lower father’s education level. The adolescents in non-depressed group were more likely having exercise habit (*P* = 0.00).

We also compared the social characteristics of only children vs. sibling children groups, LBC vs. non-LBC groups. The adolescents in only child family group were older (*P* = 0.00), with more from urban areas and lodging at home (both *P* values = 0.00), a higher proportion of excellent academic achievement (*P* = 0.00) and non-key class (*P* = 0.00). They were also more likely from single parent family and having a full-time mother (both *P* values = 0.00). The adolescents in sibling children family group were more likely from three generation household, with higher parents’ education level (both *P* values = 0.00) and exercise habit (*P* = 0.00). In addition, the adolescents in non-LBC group were older (*P* = 0.00), with more females (*P* = 0.007) and lodging at home (*P* = 0.00), with higher proportion of excellent academic achievement and studying in key class (both *P* values = 0.00). The adolescents in non-LBC group were more likely from urban areas and only child family (both *P* values = 0.00), with higher parents’ education level (both *P* values = 0.00) and exercise habit (*P* = 0.00). The adolescents in LBC group were more likely from three-generational household (*P* = 0.021). The results were presented in Additional file 1 Table S1 and Additional file 2 Table S2.

### Factors associated with depressive symptoms among rural and urban samples

32.47% of rural students and 35.11% of urban students display depressive symptoms. Although there are lots of differences of sociodemographic characteristics in rural and urban areas, the proportion of depression symptoms in rural and urban students is not significantly different. However, factors associated with depressive symptoms among rural and urban samples are totally different. The protective factors of depression in urban students are exercise habit, younger age, key class, better academic achievement and males, while LBC, poor academic achievement and females had higher risk for depression in rural area. These results are presented in Table 3 via univariate and multivariate logistic regression.

**Table 3:** Univariate and multivariate logistic regression analysis for depression in junior middle students from urban area (Wuhan city) and rural area (Jianli county)

Predictors	Univariate analysis (Urban)			Multivariate analysis (Urban)			Univariate analysis (Rural)			Multivariate analysis (Rural)		
	OR	95% CI	*P*	OR	95% CI	*P*	OR	95% CI	*P*	OR	95% CI	*P*
Age	1.168	1.040~1.312	0.009	1.159	1.026~1.309	0.018						
Gender	1.540	1.273~1.864	0.000	1.436	1.716~1.753	0.000	1.417	1.221~1.644	0.000	1.492	1.212~1.836	0.000
Academic achievement			0.000			0.000			0.000			0.000
Excellent	ref			ref			ref			ref		
Average	1.116	0.848~1.468	0.435	1.114	0.840~1.478	0.452	1.056	0.718~1.554	0.78	1.040	0.705~1.534	0.842
Poor	2.100	1.616~2.730	0.000	2.127	1.616~2.800	0.000	1.733	1.200~2.503	0.003	1.725	1.191~2.496	0.004
Key class	0.834	0.687~1.011	0.064	0.745	0.616~0.915	0.005						
Single parent family	1.397	0.960~2.032	0.081	NI			1.484	0.963~2.287	0.074	NI		
Exercise habit	0.516	0.426~0.626	0.000	0.565	0.463~0.688	0.000						
Left behind children							1.345	1.099~1.647	0.004	1.372	1.118~1.685	0.003

OR odds ratio, CI confidence interval, NI not included

### Nomograms construction and internal validation

**Fig. 2:**
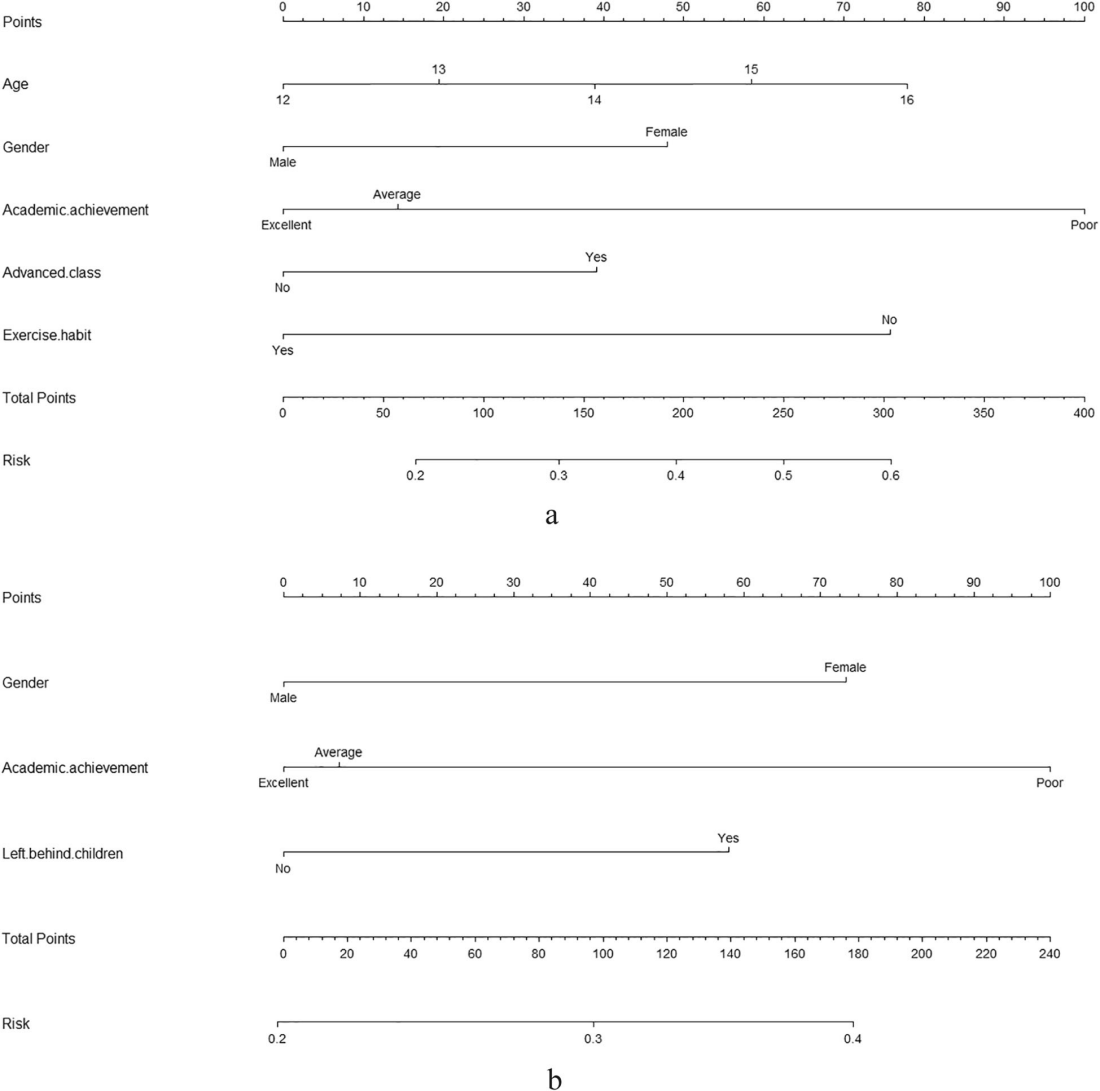
Nomograms of predicting depression of (a) urban junior middle school student and (b) rural junior middle school student

**Fig. 3:**
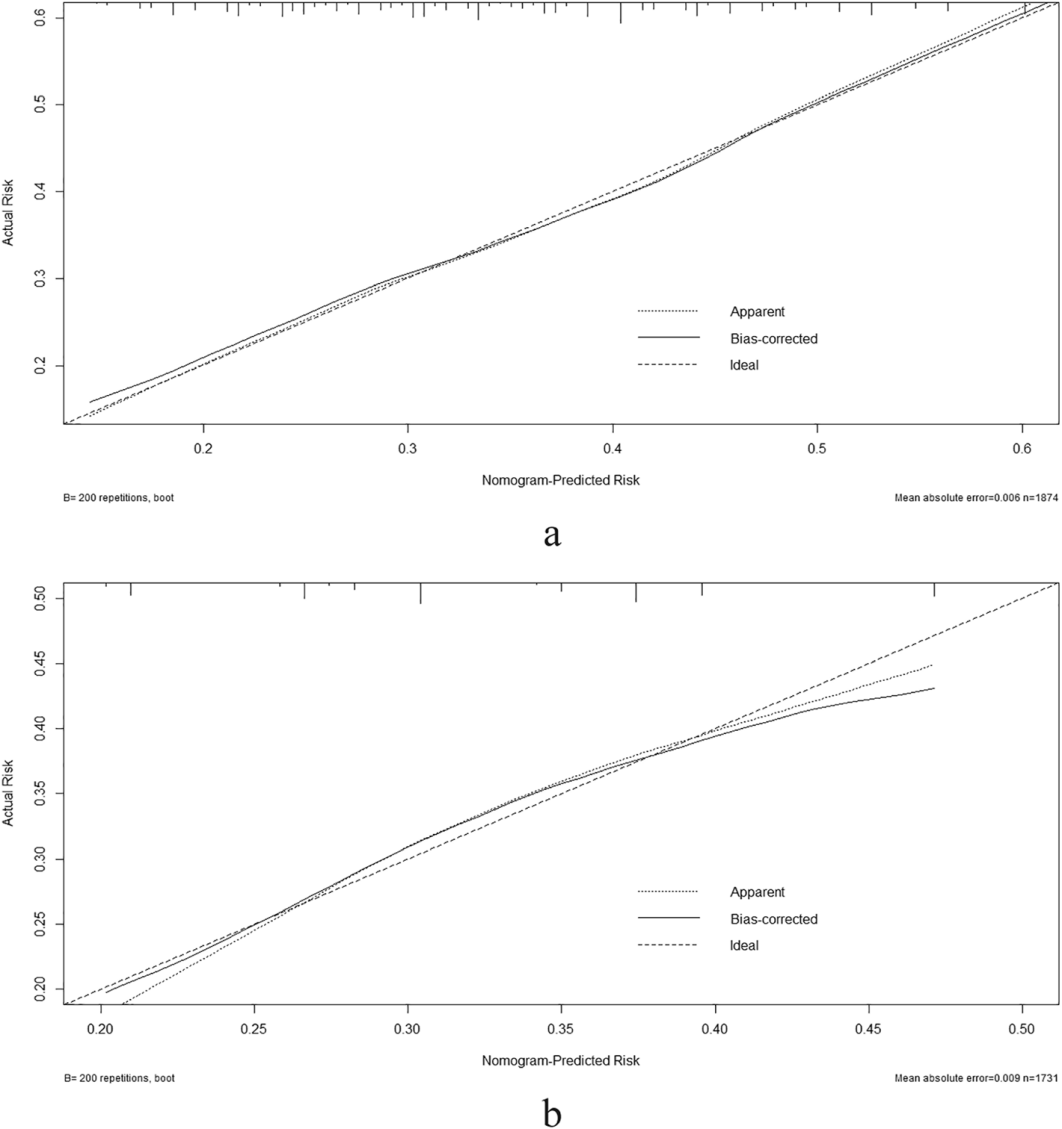
Calibration plots of the nomogram for depression prediction of the (a) urban and (b) rural junior school student. X-axis represents the nomogram-predicted probability of depression; Y-axis represents the actual depression probability. A perfectly accurate nomogram prediction model would result in a plot that the observed and predicted probabilities for given groups fall along the 45-degree line

Based on the results of the multiple logistic regression analyses, we recruited all independent prognostic factors for adolescent depression to construct the nomograms (Fig. 2). Each variable is projected upward to the value of the small ruler (Points) to get the score of each parameter. The total score was calculated by adding each score from the small ruler. The higher the total score is, the greater the likelihood of depression. The C-index of urban adolescent depression nomogram was 0.646 (95% Confidence Interval (CI), 0.639–0.653), while the C-index of rural adolescent depression nomogram was 0.600 (95% CI, 0.594–0.604). The calibration curves are plotted in Fig. 3. Dots on the plots are close to the 45° diagonal line, which suggests that the model was well calibrated.

## Discussion

Previous studies have shown the existence of various disparities between urban and rural areas in China, while Hukou-based restrictions have legitimized and endorsed institutional inequality. This cross-sectional investigation in central China also showed that there are significant differences in sociodemographic characteristics of junior middle school students in urban and rural areas. As our study showed, the proportion of only children in urban areas was much higher than that in rural areas (60.67% vs. 15.02%). In addition, 51.81% of the junior middle school students in urban areas were boys, while the proportion in rural areas was as high as 62.04%. Similarly, our data showed that there were more left-behind children, more students living in schools and the parents with lower education level in rural areas. However, the difference of depressive proportions in rural and urban students is not significant. 35.11% urban students and 32.47% rural students had depression symptoms (CES-D score ≥ 20).

Achievement and gender were found to be associated with depression symptoms for both urban and rural students. Gender factors have already been widely discussed and a considerable number of studies have examined correlations between depression and gender, suggesting that females, both adults and adolescents, have higher risk of depression than males [22]. The traditional Chinese society attaches great emphasis on education and achievements, rendering highly competitive education system [23]. Hence, the students with poor achievement tend to have lower self-esteem and more pressure on psychology. Meanwhile, some variables associated with depression symptoms in urban students were found to be different from rural students. Our study shows left-behind is associated with depression in rural students, rather than urban students. Official statistics indicate that approximately 61 million children and adolescents are left behind in rural areas, accounting for 38% of rural children in China. Approximately 70% of these left-behind children (LBC) cannot see their parents at all during a year except for holidays and festivals [24]. Considerable evidence has confirmed that children with long-term separation from parents are more likely to suffer from emotional trauma, low self-esteem, anxiety, and depression than children from intact families [25, 26]. In this study, the proportion of rural LBC in our study was 51.24%, indicating that the increasing number of LBC is becoming a social problem in rural China. We also found the LBCs are more likely from non-key classes and with higher proportion of poor achievement, and fewer LBCs have exercise habits, which were possibly due to the lack of parental supervision and education. Given the large number of LBCs and the high incidence of LBC depression, more attention and care should be given to this high-risk group of depression.

On the other hand, urban students who are in non-key class and older were more likely to be depressed, while there was no such trend among rural students. It is a unique phenomenon that junior middle school students are divided into key class and non-key class based on their entrance test scores in China [27]. This phenomenon appears more commonly in urban areas of China. Key classes mean “better students”, “better teachers” and more high-quality educational resources. Since adolescence is a susceptive period for self-esteem, potential discrimination on the non-key classes adolescents needs to be addressed.

Considerable evidences have confirmed that physical exercise can prevent and treat depression. According to WHO physical activity recommendations, children and youth aged 5–17 years old should accumulate at least 60 min of moderate to vigorous intensity physical activity daily, at least 3 times per week. In our study, the exercise habit was associated with lower risk of depression in urban adolescents, suggesting that physical activity is beneficial to the mental health of adolescents, and the importance of exercise should be more pronounced in China.

In 1979, the Chinese government started promoting and implementing the one-child policy (OCP) to ease the enomous pressure of population explosion. Previous studies have shown that one-child policy affects personality traits and depressed mood [28]. Although some of them showed that only children have elevated risk for engaging in negative psychosocial consequences, such as increasing sense of loneliness, sadness, and nervousness [29, 30], only children could possess a higher level of mental health without suffering from resource dilution [29]. In this study, there was no correlation between depression and only children. However, there were significant differences in sociodemographic characteristics of the only children and sibling children.

Numerous studies have documented that inability to recognize mental disorders reduced likelihood of help-seeking [31]. Furthermore, there is also growing evidence showing that early and correct recognition of mental disorders contributes to early treatment, and thus leads to better long-term health outcomes for those with mental disorders. The literatures offered China-based research, examining sociodemographic characteristics potentially associated with adolescent depression. Moreover, it offered effective and convenient clinical tools-nomograms, for individualized depression risk evaluation. Although nomograms have been used to provide individualized evaluation of the clinical event incidence on many occasions, the application of nomogram in screening adolescent depression is relatively rare. In the present study, two predictive nomograms included all significant independent factors which were selected by the multiple logistic regression analyses for sociodemographic characteristics in urban areas and rural areas, respectively. Guided by the nomograms, we can better predict the depression risk based on the different characteristics of urban or rural junior middle school students, which can help us to carry out identification and psychological intervention in time.

### Strengths and limitations of this study

There are several strengths in this study. This investigation is the first big-sample cross-sectional study to seek the associated risk factors for adolescent depression in urban and rural areas of Hubei province. The findings of this study helped to develop a clinical tool for screening the high-risk group of adolescent depression. Inevitably, the present study has some limitations that need to be acknowledged. First, given the limitations of the cross-sectional design, firm conclusions concerning its possible causal effects cannot be drawn. However, the findings were valuable to provide preliminary screening tool for adolescent depression. Second, regression models were derived and validated in population from Hubei Province. The generalizability to patients of other regions needs further study. Third, although we have tried to explore the influence of family economic status on the adolescent depression, the data was not objective. Since the age range of the students included in our study was just between 11 and 15 years old, most of them could not describe their family economic status accurately. These family economic data should be gathered by surveying their parents in the future.

## Conclusion

The results indicate that although there are some different sociodemographic characteristics associated with adolescent depression in rural and urban areas, the proportions of depression symptoms in rural and urban junior school students is not significantly different. The protective factors of depression in urban students are exercise habit, younger age, key class, better academic achievement and male gender, while LBC, poor academic achievement and female had higher risk for adolescent depression in rural area. Furthermore, nomograms, the effective and convenient clinical tools, were established for depression screening for junior school students.

## Supplementary information

**Additional file 1: Table S1.** Characteristics of only children and sibling children

**Additional file 2: Table S2.** Characteristics of LBC and non-LBC subjects

## Data Availability

The de-identified database used in the current study are available from the corresponding author on reasonable request.

## Abbreviations

AIC: Akaike Information Criterion
CES-D Scale: Center for Epidemiologic Studies Depression Scale
CI: Confidence Interval
C-index: Concordance Index
GDP: Gross Domestic Product
LBC: Left-behind Children
OCP: One-Child Policy
