# Supplementary material for: Sociodemographic characteristics associated with adolescent depression in urban and rural areas of Hubei province: a cross-sectional analysis
Source: BMC Psychiatry. 2019 Dec 5;19:386. doi: 10.1186/s12888-019-2380-4 (PMC6896285; doi:10.1186/s12888-019-2380-4)
Supplement: Supplementary file 1 — Additional file 1: Table S1. Characteristics of only children and sibling children [file 12888_2019_2380_MOESM1_ESM.doc]

**Table S1** Characteristics of only children and sibling children

| Variables | Only child (N=1397) | Sibling children (N=2208) | *P* |
| --- | --- | --- | --- |
| Age, years (Median, IQR) | 14 (13-14) | 13 (13-14) | 0.000 |
| Females, n (%) | 600 (42.98) | 966 (43.74) | 0.655 |
| Urban areas, n (%) | 1137 (81.39) | 737 (33.38) | 0.000 |
| Depression, n (%) | 494 (35.36) | 726 (32.88) | 0.125 |
| Accommodation, n (%) |  |  | 0.000 |
| At home | 1210 (86.67) | 1182 (53.53) |  |
| [In residence](../../../../D:/%25E5%25BA%2594%25E7%2594%25A8%25E7%25A8%258B%25E5%25BA%258F/%25E6%259C%2589%25E9%2581%2593%25E8%25AF%258D%25E5%2585%25B8/Dict/7.5.2.0/resultui/dict/javascript:%3B) | 114 (8.16) | 621 (28.13) |  |
| Others | 72 (5.15) | 405 (18.34) |  |
| Academic achievement, n (%) |  |  | 0.000 |
| Excellent | 277 (19.83) | 290 (13.13) |  |
| Average | 520 (37.22) | 802 (36.32) |  |
| Poor | 600 (42.95) | 1116 (50.54) |  |
| Key class, n (%) | 483 (34.56) | 1239 (56.12) | 0.000 |
| Three-generational household, n (%) | 966 (69.15) | 1792 (81.16) | 0.000 |
| Single parent family, n (%) | 108 (7.73) | 101 (4.57) | 0.000 |
| [Full-time](../../../../D:/%25E5%25BA%2594%25E7%2594%25A8%25E7%25A8%258B%25E5%25BA%258F/%25E6%259C%2589%25E9%2581%2593%25E8%25AF%258D%25E5%2585%25B8/Dict/7.5.2.0/resultui/dict/%3Fkeyword=full-time) mother, n (%) | 250 (17.89) | 291 (13.17) | 0.000 |
| Father's education level, n (%) |  |  | 0.000 |
| [Primary School and Below](../../../../D:/%25E5%25BA%2594%25E7%2594%25A8%25E7%25A8%258B%25E5%25BA%258F/%25E6%259C%2589%25E9%2581%2593%25E8%25AF%258D%25E5%2585%25B8/Dict/7.5.2.0/resultui/dict/javascript:%3B) | 221 (15.85) | 204 (9.23) |  |
| Secondary school | 904 (64.67) | 1023 (46.31) |  |
| College degree and above | 272 (19.47) | 981 (44.45) |  |
| Mother's education level, n (%) |  |  | 0.000 |
| [Primary School or Below](../../../../D:/%25E5%25BA%2594%25E7%2594%25A8%25E7%25A8%258B%25E5%25BA%258F/%25E6%259C%2589%25E9%2581%2593%25E8%25AF%258D%25E5%2585%25B8/Dict/7.5.2.0/resultui/dict/javascript:%3B) | 412 (29.49) | 251 (11.38) |  |
| Secondary school | 757 (54.19) | 1053 (47.69) |  |
| College degree and above | 228 (16.32) | 904 (40.94) |  |
| [Exercise habit](../../../../D:/%25E5%25BA%2594%25E7%2594%25A8%25E7%25A8%258B%25E5%25BA%258F/%25E6%259C%2589%25E9%2581%2593%25E8%25AF%258D%25E5%2585%25B8/Dict/7.5.2.0/resultui/dict/javascript:%3B), n (%) | 639 (45.74) | 1146 (51.90) | 0.000 |
