# Supplementary material for: Sociodemographic characteristics associated with adolescent depression in urban and rural areas of Hubei province: a cross-sectional analysis
Source: BMC Psychiatry. 2019 Dec 5;19:386. doi: 10.1186/s12888-019-2380-4 (PMC6896285; doi:10.1186/s12888-019-2380-4)
Supplement: Supplementary file 2 — Additional file 2: Table S2. Characteristics of LBC and non-LBC subjects [file 12888_2019_2380_MOESM2_ESM.doc]

**Table S2** Characteristics of LBC and non-LBC subjects

| Variables | LBC (N= 1401) | Non-LBC (N=2564) | *P* |
| --- | --- | --- | --- |
| Age, years (Median, IQR) | 13 (12-14) | 14 (13-14) | 0.000 |
| Females, n (%) | 557 (39.77) | 1146 (44.70) | 0.007 |
| Urban areas, n (%) | 154 (10.99) | 1720 (67.08) | 0.000 |
| Depression, n (%) | 506 (36.12) | 844 (32.92) | 0.066 |
| Accommodation, n (%) |  |  | 0.000 |
| At home | 444 (31.69) | 2063 (80.46) |  |
| [In residence](../../../../D:/%25E5%25BA%2594%25E7%2594%25A8%25E7%25A8%258B%25E5%25BA%258F/%25E6%259C%2589%25E9%2581%2593%25E8%25AF%258D%25E5%2585%25B8/Dict/7.5.2.0/resultui/dict/javascript:%3B) | 585 (41.76) | 300 (11.70) |  |
| Others | 372 (26.55) | 201 (7.84) |  |
| Academic achievement, n (%) |  |  | 0.000 |
| Excellent | 143 (10.21) | 461 (17.98) |  |
| Average | 545 (38.90) | 917 (35.76) |  |
| Poor | 713 (50.89) | 1186 (46.26) |  |
| Key class, n (%) | 404 (28.84) | 1247 (48.63) | 0.000 |
| Three-generational household, n (%) | 1108 (79.09) | 1935 (75.47) | 0.021 |
| Only children, n (%) | 279 (19.91) | 1190 (46.41) | 0.000 |
| Single parent family, n (%) | 90 (6.42) | 143 (5.58) | 0.318 |
| [Full-time](../../../../D:/%25E5%25BA%2594%25E7%2594%25A8%25E7%25A8%258B%25E5%25BA%258F/%25E6%259C%2589%25E9%2581%2593%25E8%25AF%258D%25E5%2585%25B8/Dict/7.5.2.0/resultui/dict/%3Fkeyword=full-time) mother, n (%) | 199 (14.20) | 431 (16.81) | 0.055 |
| Father's education level, n (%) |  |  | 0.000 |
| [Primary school or below](../../../../D:/%25E5%25BA%2594%25E7%2594%25A8%25E7%25A8%258B%25E5%25BA%258F/%25E6%259C%2589%25E9%2581%2593%25E8%25AF%258D%25E5%2585%25B8/Dict/7.5.2.0/resultui/dict/javascript:%3B) | 199 (19.40) | 277 (10.80) |  |
| Secondary school | 970 (69.24) | 1354 (52.81) |  |
| College degree or above | 159 (11.35) | 933 (36.39) |  |
| Mother's education level, n (%) |  |  | 0.000 |
| [Primary school or below](../../../../D:/%25E5%25BA%2594%25E7%2594%25A8%25E7%25A8%258B%25E5%25BA%258F/%25E6%259C%2589%25E9%2581%2593%25E8%25AF%258D%25E5%2585%25B8/Dict/7.5.2.0/resultui/dict/javascript:%3B) | 499 (35.62) | 439 (17.12) |  |
| Secondary school | 783 (55.89) | 1281 (49.96) |  |
| College degree or above  Exercise habit, n (%) | 118 (8.42)  580 (41.40) | 844 (32.92)  1304 (50.86) | 0.000 |
